# Expression of cystatin C in the female reproductive tract and its effect on human sperm capacitation

**DOI:** 10.1186/s12958-018-0327-0

**Published:** 2018-01-30

**Authors:** Robert Kuo-Kuang Lee, Huan-Chin Tseng, Yuh-Ming Hwu, Chi-Chen Fan, Ming-Huei Lin, Jhih-Jie Yu, Ling-Yu Yeh, Sheng-Hsiang Li

**Affiliations:** 1 Department of Medical Research, Mackay Memorial Hospital, Tamsui District, New Taipei City, 251 Taiwan; 2 Department of Obstetrics and Gynecology, Mackay Memorial Hospital, Taipei City, 104 Taiwan; 3 Department of Obstetrics and Gynecology, Taipei Medical University, Taipei City, 110 Taiwan; 4 Mackay Medical College, Sanzhi District, New Taipei City, 252 Taiwan; 5 Mackay Junior College of Medicine, Nursing, and Management, Beitou District, Taipei City, 112 Taiwan; 6 Office of Superintendent, Mackay Memorial Hospital, Taipei City, Taiwan; 7 Department of Medical Laboratory Science and Biotechnology, Yuanpei University, Hsinchu, 300 Taiwan

## Abstract

**Background:**

Cystatin C (CST3), a cysteine protease inhibitor in seminal plasma, is expressed in animal uteri. However, its expression in the human female reproductive tract and its effect on human sperm capacitation are unclear.

**Methods:**

The cellular localization of CST3 was observed using immunohistochemistry. The binding of CST3 to sperm was examined using immunocytochemistry. Sperm motility parameters were analyzed using computer-assisted sperm analysis. Sperm capacitation was evaluated by analyzing cholesterol content, protein tyrosine phosphorylation levels, and the acrosome reaction.

**Results:**

Immunohistochemical staining demonstrated that CST3 is prominently expressed in the female reproductive tract, including the epithelial lining and cervix and endometrium fluids, particularly at times near ovulation. It can bind to human sperm on the post-acrosomal head region and the mid and principal piece of the tail. CST3 enhances sperm motility and inhibits the signal initiating sperm capacitation, i.e., efflux of cholesterol from the sperm plasma membrane and a late sperm capacitation event, i.e., the increase in the sperm protein tyrosine phosphorylation. The suppressive trend on sperm acrosome reaction further supports CST3’s ability to inhibit sperm capacitation.

**Conclusions:**

These findings suggest that cervical CST3 may prevent precocious capacitation and acrosome reaction, thus preserving sperm fertilizing ability before it reaches the fallopian tube. Additionally, CST3 may help sperm enter the upper reproductive tract by enhancing sperm motility.

## Background

Cystatin C (CST3), a small secretory protein encoded by the *CST3* gene, is extensively expressed in various tissues and body fluids and possesses potent inhibitory activity against cysteine proteases [1]. CST3 level is high in blood serum and is removed from the bloodstream via the kidney’s glomerular filtration; thus, it is a simple, accurate, and rapid endogenous marker of glomerular filtration rate [2, 3].

CST3 is prominently expressed in the male reproductive tract with high levels in seminal plasma, approximately 37-fold higher than in serum [4, 5]. The prostasome, an exosome-like vesicle in seminal plasma secreted by the prostate epithelium [6], can inhibit sperm capacitation [7, 8]; CST3 was demonstrated to be associated with the prostasome [9]. Thus, CST3 may be important in sperm physiology too.

Ejaculated sperm must reside in the female reproductive tract to be able to fertilize an egg, known as capacitation first described and defined in the early 1950s [10, 11]. The sperm’s surface is exposed to protein-rich secretions from the seminal plasma, cervix, uterus, or fallopian tubes during transit. Thus, proteins secreted by these tissues interact with and modify changes in sperm physiology to make fertilization possible [12, 13]. Capacitation is a physiologically complex change and may be initiated by cholesterol removal from the sperm plasma membrane, leading to changes in the membrane’s structure and fluidity and increasing the sperm’s calcium and bicarbonate ion permeability [14, 15]. This activates a cyclic AMP (cAMP)-dependent protein kinase and induces tyrosine phosphorylation of a subset of sperm proteins [16, 17].

Seminal plasma, a mix of male accessory sexual gland secretions, has been demonstrated to reverse sperm capacitation [18]. The affecting factor, defined as decapacitation factor (DF), is removed from the sperm surface before or during capacitation [13]. Previous studies have reported that potential DFs are primarily present in human and rodent seminal plasma [19–29]. However, as human sperm enters the cervix, it must migrate through interstices formed by cervical mucus [30]. The interstices are smaller than the sperm head; thus, proteins derived from seminal plasma and bound to sperm surface are potentially stripped from sperm when it passes the cervix because seminal proteins are speculated to weakly associate with sperm membrane [31]. This seemingly suggests that seminal plasma DFs become effective before sperm enters the cervix. How sperm can preserve its fertility before reaching the fertilization site, i.e., the ampulla of the fallopian tube, warrants further studies. Previous studies have evaluated the importance of oviductal components like hyaluronic acid [32] and some oviductal proteins [33, 34] in the female reproductive tract that contribute to preserve sperm viability and motility until fertilization. Human sperm can prolong acrosomal integrity as it stays in the cervix for up to five days [35]. This indicates that secretory proteins in the cervix are important in preserving sperm fertility.

We believe that, except seminal DFs, some proteins secreted by the lower female reproductive tract, such as cervical mucus or uterine fluid, may protect sperm from premature capacitation and thus preserve the sperm’s fertilization ability until it reaches the fallopian tube. Previous studies have reported CST3 expression in the endometrial luminal epithelium and glandular epithelium, secreted into the uterine cavity, and upregulated by progesterone in the ovine uterus [36]. In rat uterus, CST3 was expressed in the glandular epithelium, secreted into the uterine lumen, and downregulated by estrogen during the peri-implantation period [37]. In pigs, *CST3* mRNA levels increased in the endometrial epithelium during pregnancy, whereas progesterone decreased CST3 expression in the epithelium but increased it in the stromal compartment [38]. However, CST3 cellular distribution in the human female reproductive tract has not been explored. Here, we investigated the cellular localization of CST3 in the human female reproductive tract and its effect on human sperm capacitation.

## Methods

### Samples and ethics

Formalin-fixed and paraffin-embedded tissues, including the cervix, uterine endometrium, and fallopian tubes in the proliferative and secretory phases, were obtained from the Department of Pathology, Mackay Memorial Hospital (Taipei, Taiwan). Patients with the following features were excluded: > 40 or < 20 years old, menstruation irregular or regular with hormone treatment, a malignant disease requiring surgery, and systemic disease. Semen samples were collected via masturbation after 2–3 days of sexual abstinence. After liquefaction for at least 30 min, routine semen analysis was conducted according to the 2010 World Health Organization criteria [39]. Semen samples with > 40% motility and sperm counts > 15 million/ml were included. Clinical paraffin section and semen usage was reviewed and approved by the hospital’s institutional review board (approval number 15MMHIS101).

### Immunohistochemical staining

An automatic staining machine (BenchMark XT; Ventana Medical Systems, Tucson, AZ, USA) was used for immunohistochemical staining using the XT ultraView (UV) 3, 3′-diaminobenzidine (DAB) detection kit (Ventana Medical Systems). Tissue sections (4 μm) on slides were deparaffinized, hydrated, and heated to induce antigen retrieval according to a previously described method [40]. After inactivating endogenous peroxidase activity using a UV inhibitor, the sections were incubated with a rabbit monoclonal anti-CST3 antibody (ab109508; Abcam, Cambridge, MA, USA) or a control antibody (1:5000) in a blocking solution at 37 °C for 30 min. Sections were rinsed and then incubated with UV horseradish peroxidase (HRP) at 37 °C for 8 min. Staining signals were developed using UV DAB and hydrogen peroxide at 37 °C for 8 min. Slides were finally incubated with UV copper for 4 min to enhance signal intensity, counterstained with hematoxylin (Vector Laboratories, Burlingame, CA, USA), and photographed using TissueFaxs software on an automated acquisition system (TissueGnostics, Vienna, Austria).

### Human sperm preparation

Sperms were prepared using the swim-up method. Briefly, 1.0 ml liquefied semen was mixed with 6 ml 1X diluted Earle’s balanced salt (E-7510; Sigma-Aldrich, St. Louis, MO, USA) medium supplemented with 0.3 mM sodium pyruvate (hereafter abbreviated as EBSS). After centrifuging at 500 *g* for 10 min, approximately 0.2 ml of the lowest layer was collected, resuspended, and transferred to an Eppendorf tube containing 1 ml EBSS medium. The tube was set at a 45° angle to make sperm swim-up in a 5% (*v*/v) CO_2_ atmosphere in humidified air for 30 min. Sperm with progressive motility were collected from the upper layer.

Highly motile sperm was purified using two-layer PureSperm (Nidacon International AB, Mölndal, Sweden) discontinuous density gradient centrifugation. Briefly, 1.0 ml liquefied semen was layered above the upper layer of the PureSperm gradient solution prepared by layering 1.0 ml PureSperm40 on 1.0 ml PureSperm80 solution. After centrifuging at 300 *g* for 20 min, the upper supernatant was removed except the lowest 0.5 ml and resuspended in 5 ml EBSS medium. After washing with EBSS by centrifugation again, the lowest 0.1 ml was collected. The sperm’s working concentration could be arranged by centrifugation and then be resuspended in a suitable volume of medium.

### Immunolocalization of CST3 on human sperm

To evaluate whether CST3 is a sperm-binding protein, sperm isolated using the swim-up method was smeared on slides, fixed in 4% (*w*/*v*) paraformaldehyde, allowed to air-dry, and washed twice with phosphate-buffered saline (PBS). The slides were incubated in a blocking solution, PBS containing 10% (*v*/v) normal goat serum, for 1 h at room temperature and then incubated with the anti-CST3 antibody (1:250; ab109508; Abcam) or control antiserum (1:100 in blocking solution) for 1 h. After repeatedly washing thrice with PBS for 5 min each to remove excess antibodies, the slides were incubated with fluorescein isothiocyanate (FITC)-conjugated goat anti-rabbit IgG (Vector Laboratories, 1:500 in blocking solution) for 40 min. The slides were then washed using previously described procedure, counterstained with 5 μg/ml Hoechst 33,258, and mounted in Prolong Gold antifade medium (Invitrogen Molecular Probes, Eugene, OR, USA) after briefly rinsing with PBS. The staining signal was visualized using an epifluorescence microscope equipped with a digital camera (Olympus DP 71, Tokyo, Japan).

To evaluate whether exogenous human CST3 binds to human sperm, 2 μg/ml CST3 (PRO-413; ProSpec, East Brunswick, NJ, USA) was incubated with living sperm (10^6^ cells/ml, isolated using PureSperm density gradient centrifugation) in Eppendorf tubes at 37 °C for 20 min. The unbound CST3 was discarded using PBS by centrifuging at 600 *g* for 5 min at room temperature. Sperm was then transferred onto slides, fixed in 4% (*w*/*v*) paraformaldehyde for 20 min at room temperature, and air-dried.

### Analysis of sperm motility

Human sperm (2 × 10^7^ cells/ml) isolated using PureSperm density gradient centrifugation was incubated in 100 μl EBSS medium containing sodium bicarbonate (10 mM) and bovine serum albumin (BSA, 3 mg/ml) at 37 °C under 5% (*v*/v) CO_2_ in humidified air. To analyze CST3 effect on sperm motility, the medium was supplemented with or the medium’s BSA was replaced with 0.25 mg/ml CST3. Sperm motility was determined using a computer-assisted sperm analysis system with a sperm motility analyzer (IVOS version 10; Hamilton-Thorne Research, Beverly, MA, USA). A 3 μl sample was loaded into a 20 μm deep Leja slide at 37 °C for analyses at 30 min intervals up to 180 min. Motility parameters, including averaged path velocity (VAP), straight-line velocity (VSL), curvilinear velocity (VCL), percentages of sperm motility (VAP > 7 μm/s), and progressive motility [VAP > 50 μm/s and path straightness (STR) > 80%, where STR = VSL/VAP] were measured. Analyzer settings were to default values. For each assay, five fields with 100–400 sperm were analyzed.

### Cholesterol efflux assay

Human spermatozoa (2 × 10^6^ cells/ml) were preincubated with or without CST3 (0.125, 0.25, or 0.5 mg/ml) in 100 μl EBSS medium for 20 min and then supplemented with BSA (3 mg/ml) or sodium bicarbonate (10 mM) for capacitation at 37 °C for 3 h under 5% (*v*/v) CO_2_ in humidified air. The sperm was then centrifuged at 20,000 *g* to obtain the pellet. Cholesterol on sperm plasma membrane was extracted following a previously described method [41]. Cholesterol content was measured using an Amplex Red Cholesterol Assay Kit (Invitrogen Molecular Probes) according to manufacturer instructions. A cholesterol standard curve was prepared using the cholesterol reference standard provided with the kit to calculate the cholesterol content of the samples.

### Evaluating capacitation-associated sperm tyrosine phosphorylation

Capacitation-accompanied increases in 105- and 80-kDa human sperm proteins tyrosine phosphorylation were examined according to a previously described method [42]. Briefly, approximately 2 × 10^6^ sperm/ml were incubated in 100 μl EBSS medium with or without CST3 (0.125, 0.25 or 0.5 mg/ml) at 37 °C for 20 min; BSA and sodium bicarbonate were then added as described for in vitro capacitation. After 3 h of incubation, the soluble sperm protein extract fractions were subjected to 10% SDS-PAGE. Proteins on the gel were electrotransferred onto nitrocellulose. Western blots were performed using anti-phosphotyrosine (clone 4G10; Upstate Biotechnology, Lake Placid, NY, USA) and α-tubulin antibody (Sigma-Aldrich). Relative expression levels were normalized by the loading control levels, i.e., α-tubulin protein.

### Evaluation of the acrosome reaction

PureSperm-isolated sperm (2 × 10^6^ sperm/ml) were incubated with or without CST3 (0.0625, 0.125, 0.25, or 0.5 mg/ml); subsequently, capacitation was performed as described above. The capacitated sperm was then treated with or without 10 μM A23187 in 0.1% (*v*/v) dimethyl sulfoxide at 37 °C for 30 min and was collected by centrifugation at 600 *g* for 5 min, smeared onto slides, and air-dried. The slides were immersed in methanol for 1 min, washed thrice in PBS for 5 min each, and stained with 10 μg/ml tetramethylrhodamine isothiocyanate-conjugated peanut agglutinin lectin (PNA; Sigma-Aldrich) in the dark for 30 min. After washing the slides thrice in PBS for 5 min each, sperm was counterstained with Hoechst 33,258 (5 μg/ml) for 30 s, mounted with Prolong Gold antifade medium (Invitrogen Molecular Probes), and examined with a fluorescence microscope (BX 40, Olympus). A random sample of 500 sperm cells per group was evaluated.

### Statistical analysis

Data are presented as mean ± SD. Differences were analyzed by one-way ANOVA followed by the Bonferroni post-hoc test using GraphPad software (GraphPad, San Diego, CA, USA). *p* < 0.05 was considered statistically significant.

## Results

### CST3 expression in the female reproductive tract

**Fig. 1:**
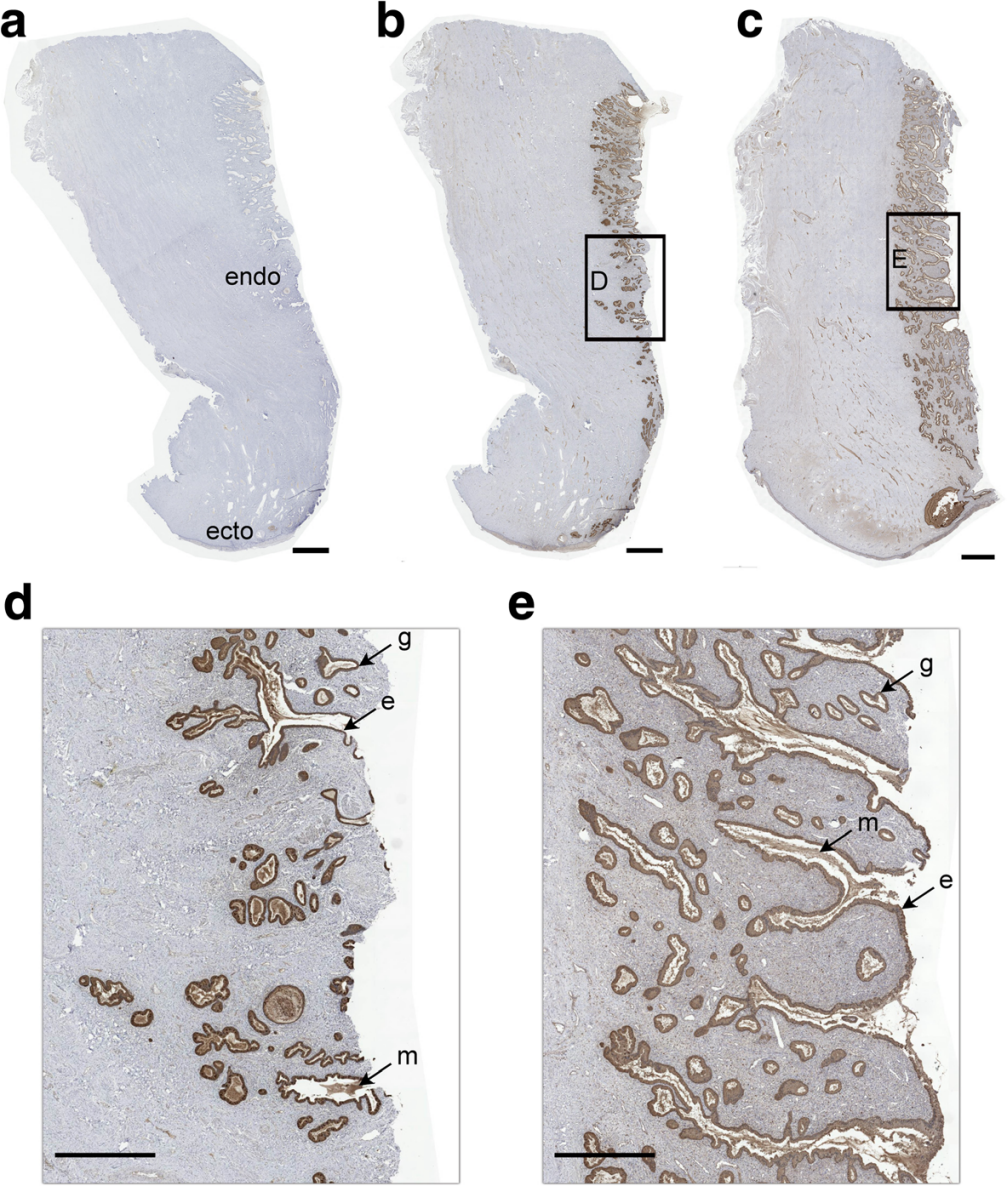
CST3 immunolocalization in the cervix. Paraffin sections of the cervix at early (**a** and **b**) and late (**c**) proliferative phases of the menstrual cycle were immunostained using control antibody (**a**) or anti-CST3 antibody (**b** and **c**). The selected region in **b** and **c** was enlarged for clarity (**d** and **e**). The staining signals were colored brown by the HRP substrate DAB. Symbols: ecto, ectocervix; endo, endocervix; e, cervical epithelium; g, cervical gland; m, cervical mucus. Bars: A–C, 2 mm; D and E, 1 mm

**Fig. 2:**
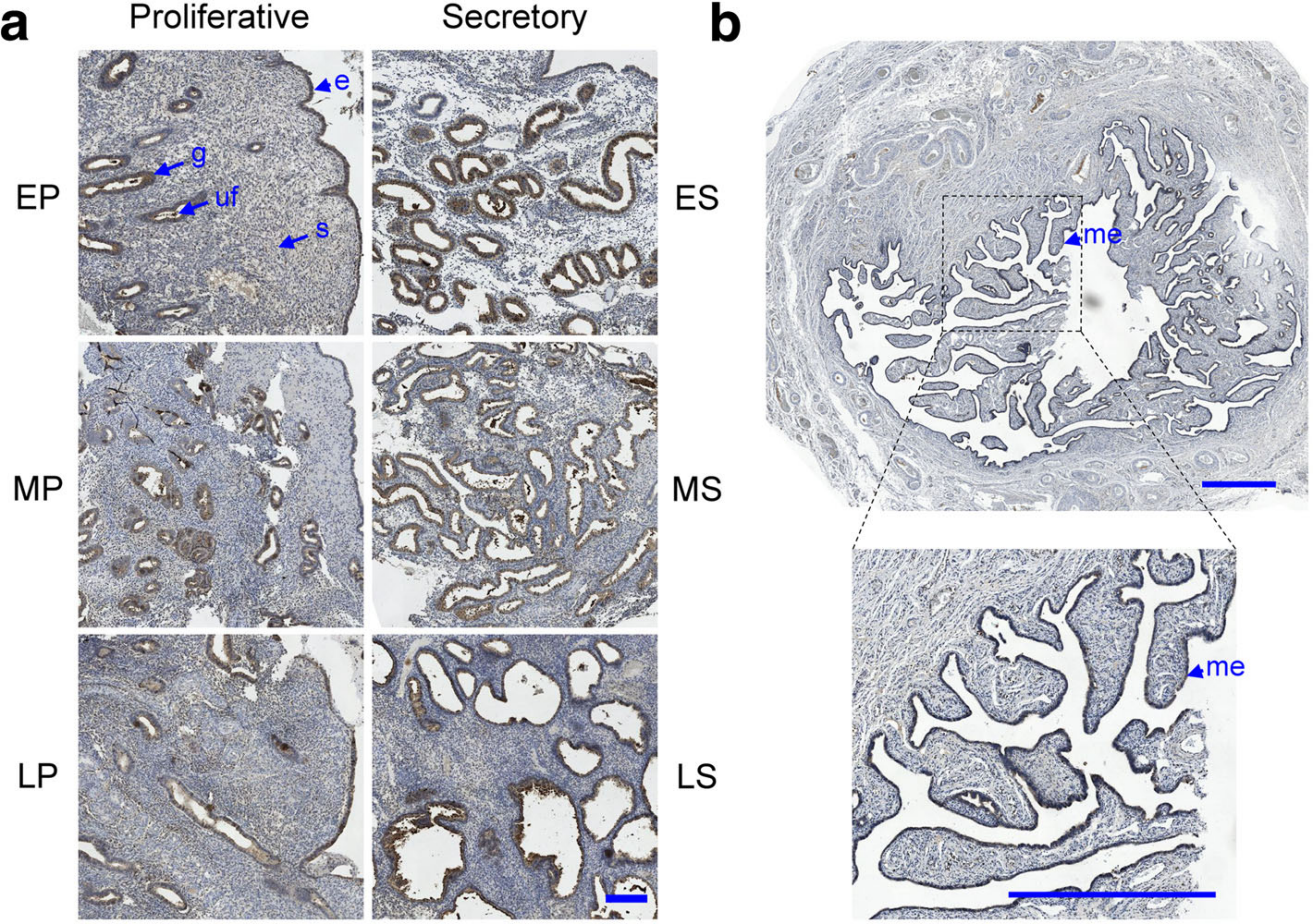
CST3 expression and localization in the cyclic endometrium and fallopian tube. Paraffin sections prepared by the endometrial curettage during various proliferative and secretory phases (**a**) and the fallopian tube (**b**) were incubated with anti-CST3 antibody as stated in the text. Symbols: e, epithelium; g, gland; me, mucosal epithelium; s, stroma; uf, uterine fluid. The menstrual cycle is divided into six subphases: EP, early proliferative; MP, mid-proliferative; LP, late-proliferative; ES, early secretory; MS, mid-secretory; and LS, late-secretory phase. Bar: 200 μm in A, 1 mm in B

The cellular localization of CST3 in the female reproductive tract was observed using immunohistochemical staining. Contrary to control antiserum (Fig. 1A), CST3 was prominently detected in the endocervical canal during mid- (Fig. 1B) and late-proliferative phases (Fig. 1C) of the menstrual cycle. CST3 was detected in cervical epithelial cells, glands, and mucus (Figs 1D and E), particularly during the late-proliferative phase (Fig. 1E). In the uterus, CST3 was expressed in the luminal and glandular endometrial epithelial cells throughout the proliferative and luteal menstrual cycle phases. CST3 was also detected in uterine fluid, indicating that it could be expressed and secreted by the endometrium (Fig. 2A) and in the fallopian tube epithelial cells, although its expression levels were lower there (Fig. 2B).

### Binding of CST3 to human sperm

**Fig. 3:**
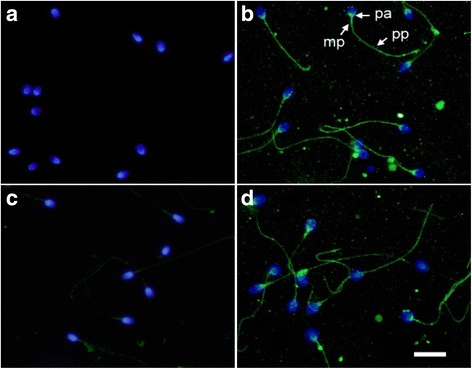
CST3 binding to human sperm. Sperm isolated using the swim-up method was smeared onto slides for immunohistochemical staining (**a** and **b**). Sperm isolated using PureSperm density gradient were incubated without (**c**) or with (**d**) CST3. After washing off unbound CST3, sperm was transferred onto slides and fixed with 4% (*w*/*v*) paraformaldehyde. Slides were incubated with control antiserum (**a**) or anti-CST3 antibody (**b**–**d**) diluted 1:250, treated with FITC-conjugated goat anti-rabbit IgG, and then counterstained with Hoechst dye to localize the nuclei for contrast. Symbols: mp, midpiece; pa, post-acrosome; pp., principal piece. Bar: 10 μm

To examine whether CST3 binds to human sperm, sperm isolated from liquefied semen using the swim-up method was immunostained using anti-CST3 antibody or control antiserum. Contrary to control slides stained using control antiserum (Fig. 3A), CST3 was primarily detected on the post-acrosomal head region and the mid and principal piece of the tail (Fig. 3B). When sperm was isolated using PureSperm density gradient centrifugation, CST3 staining was very weak with only a faint signal on the sperm tail (Fig. 3C). However, CST3 was prominently stained on sperm with the same binding pattern when human sperm was incubated with exogenous CST3 (Fig. 3D), indicating that CST3 can bind to human sperm.

### Enhancement of sperm motility by CST3

**Fig. 4:**
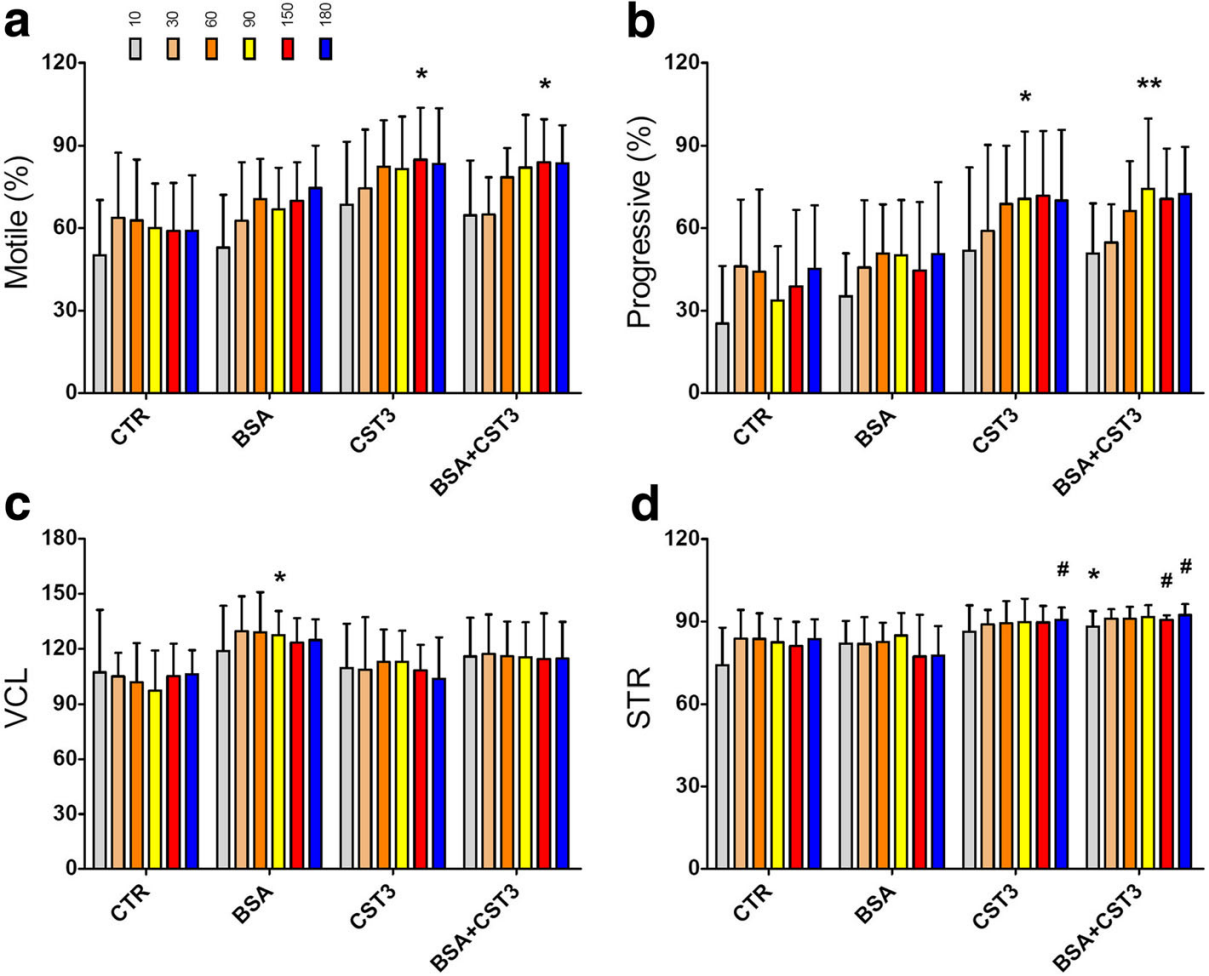
Effects of CST3 on human sperm motility. Sperm isolated by density gradient was incubated in EBSS medium, or the medium was supplemented with BSA, CST3, or BSA and CST3 (see text for details) at 37 °C under 5% (*v*/v) CO_2_ for 3 h. Motility parameters, including motile sperm percentage (**a**), progressively motile sperm percentage (**b**), VCL (**c**), and STR (**d**), at each specified incubation time were determined. Points are expressed as mean ± SD of eight independent determinations. *p < 0.05 and **p < 0.01 vs. the control (CTR) group; #*p* < 0.05 vs. the BSA group at corresponding time points

Human sperm isolated using density gradient centrifugation was mobile with a visibly beating tail in EBSS medium with sodium bicarbonate (the control group). When the medium was supplemented with BSA, a higher percentage of motile sperm was observed than in the control group. Interestingly, when supplementing the medium with 0.125 mg/ml CST3, the motile sperm percentage was comparable with the BSA group (not shown). However, 0.25 mg/ml CST3 resulted in a relatively higher motile sperm percentage than medium with BSA (Fig. 4A). Increasing CST3 concentration to 0.6 mg/ml had similar results (not shown). Also, co-incubation with CST3 and BSA showed an increased level similar to the CST3-treated group (Fig. 4A). The percentage of progressively motile sperm showed a similar tendency, with relatively higher percentages in the CST3 or in the CST3 and BSA co-treated group (Fig. 4B). A higher VCL was found in the BSA group than in the other three groups, with the CST3 group showing a relatively lower VCL (Fig. 4C). Additionally, STR is higher in sperm treated with CST3, and a considerable STR percentage is still maintained even by co-treatment with BSA (Fig. 4D). Taken together, CST3 can enhance sperm motility and maintain linear motility.

### Inhibition of sperm capacitation by CST3

**Fig. 5:**
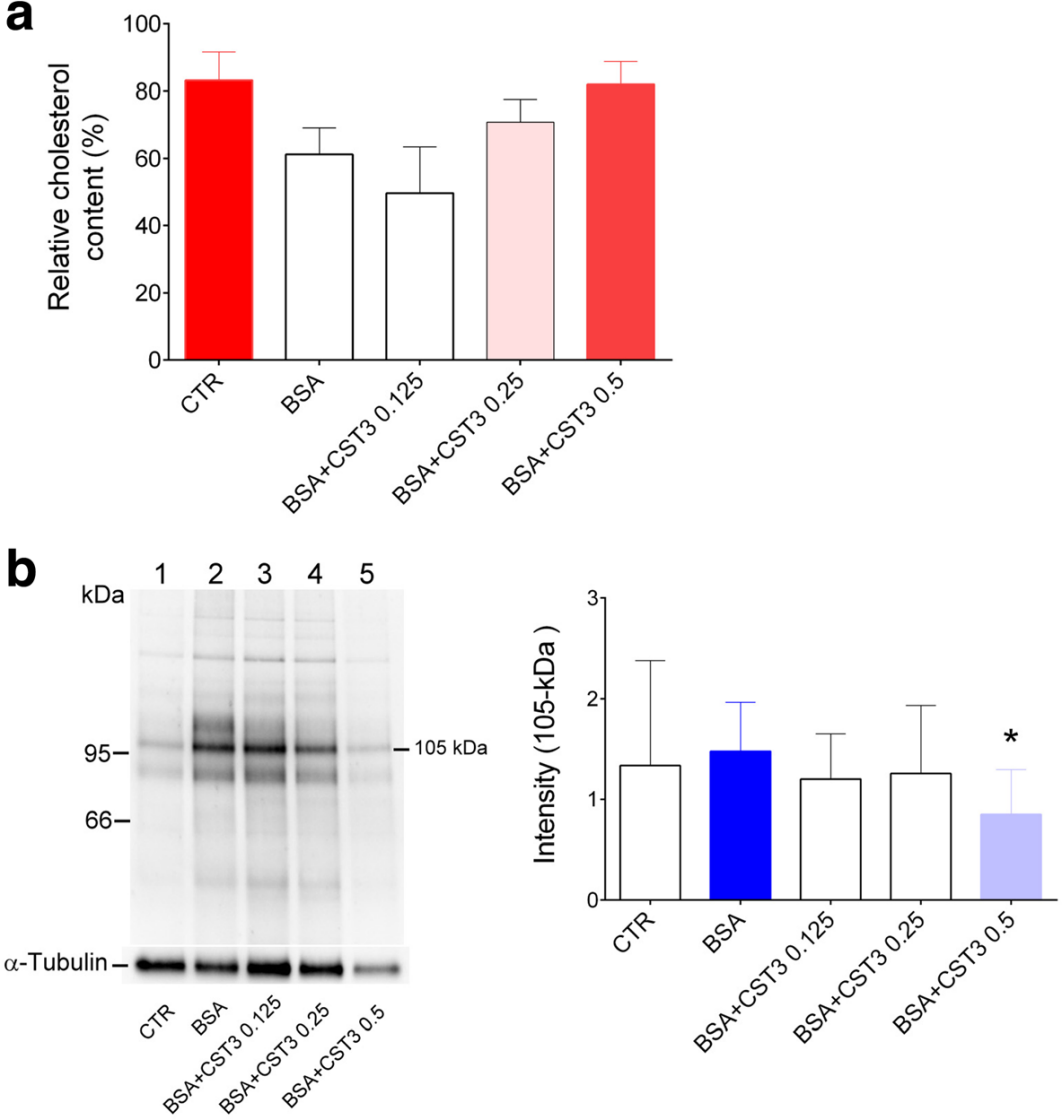
CST3 inhibits sperm capacitation. Human sperm cultured in EBSS medium was preincubated with or without CST3 (0.125, 0.25, or 0.5 mg/ml) for 20 min, followed by incubation with BSA (3 mg/ml) and sodium bicarbonate (10 mM) for 3 h to induce sperm capacitation. **a** Cholesterol retained on the sperm was measured. Data are expressed as mean ± SD of three independent experiments. **b** Phosphorylation levels of sperm protein’s tyrosine residues. A representative anti-phosphotyrosine western blot, out of six experiments, is shown. The relative intensity of the 105-kDa band was quantified using ImageJ, and all values were normalized to the loading control, α-tubulin. *p < 0.05 vs. medium supplemented with BSA and bicarbonate

We assessed CST3 effects on sperm capacitation because of its sperm-binding ability and its effect on sperm motility, which was still linear even in the presence of BSA. Cholesterol efflux is a key event initiating sperm capacitation. Thus, we examined CST3 influence on albumin-induced capacitation-associated cholesterol efflux. As expected, BSA induced cholesterol efflux from the capacitated sperm; thus, lower cholesterol contents were retained on capacitated sperm rather than uncapacitated control sperm. However, CST3 suppressed cholesterol efflux in a dose-dependent, statistically insignificant manner (Fig. 5A), suggesting that CST3 might suppress sperm capacitation by blocking cholesterol efflux from the sperm plasma membrane.

To evaluate CST3 effects on downstream sperm capacitation signaling, the characteristic sperm protein tyrosine phosphorylation pattern was examined. Both bicarbonate and BSA are required for sperm protein tyrosine phosphorylation induction [43]. As expected, the control medium showed basal phosphorylation levels, while BSA remarkably increased the phosphorylation levels. CST3 significantly attenuated the induction at a concentration of 0.5 mg/ml (Fig. 5B).

### CST3 inhibits sperm acrosome reaction

**Fig. 6:**
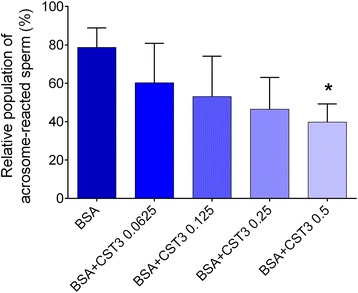
CST3 suppresses sperm acrosome reaction. Human sperm was capacitated as stated in the text. The sperm acrosome reaction induced by the calcium ionophore A23187 was evaluated using PNA staining. Minimum 500 sperm cells were evaluated per trial. Data are expressed as mean ± SD of five independent experiments. *p < 0.05 vs. medium supplemented with BSA and bicarbonate

Only capacitated sperm can be induced to undergo acrosome reaction [13]; thus, if CST3 inhibits sperm capacitation, it might suppress the acrosome reaction. We therefore examined whether CST3 has inhibitory effects on sperm acrosome reaction induced by the calcium ionophore A23187. As shown in Fig. 6, BSA-capacitated sperm showed remarkable enhancements in acrosome reaction after A23187 induction; contrarily, the percentage of acrosome-reacted sperm gradually attenuated with significant inhibition when sperm was co-incubated with CST3 and BSA at a concentration of 0.5 mg/ml. These findings suggest that CST3 can inhibit sperm capacitation induced in vitro.

## Discussion

CST3 is highly expressed in the male genital tract and semen [4, 5]. Although CST3 is expressed in the uterus of rats, sheep, and pigs [36–38], its expression in the human female genital tract is not explored. Here, we reveal the CST3 expression and localization in the cervix, uterus, and fallopian tube. CST3 was prominently expressed in the cervix and endometrium epithelia cells; however, CST3 detection in the cervical mucus and uterine fluid near ovulation is more important. The elevated serum estrogen concentration during the mid- and late-proliferative phases of the menstrual cycle leads to the cervical mucus becoming clear and slippery [44], allowing sperm to enter the cervix. CST3 expression in the cervix creates conditions for interaction between the protein and sperm.

Seminal proteins may be removed from sperm surface during transit into the cervix [31, 45]. Only sperm with a normal morphology and progressive motility can penetrate the cervix [45, 46]. It is guided into the cervical crypts by cervical mucus strands [39], retained up to several days [35], and gradually released into the upper female reproductive tract.

PureSperm density gradient centrifugation removed immature, less mobile, damaged sperm, and seminal plasma [47, 48]; thus, more morphologically normal and progressively motile sperm can be prepared [49]. Sperm isolated using PureSperm density gradient centrifugation may be similar to sperm entering the cervix and can be used to evaluate interactions between sperm and cervical mucus and/or uterine fluid.

CST3 originating from seminal plasma might bind to human sperm, as demonstrated by immunostaining sperm isolated from semen using the swim-up method; however, seminal CST3 is removed from sperm surface after PureSperm density gradient centrifugation. CST3 might bind to sperm again, as exogenous CST3 was incubated with sperm isolated using density gradient separation. These results indicate that CST3 is a sperm-binding protein and can be stripped from PureSperm-isolated sperm. Thus, PureSperm-isolated sperm was used to mimic sperm that entered the cervix for evaluating cervical CST3 effects on sperm physiology. CST3 tended to inhibit sperm capacitation initiation signaling, i.e., cholesterol efflux from the sperm plasma membrane and a late sperm capacitation event, i.e., increase in sperm protein tyrosine phosphorylation.

Cervical secretions provide hospitable environments for sperm accommodation [45]. Sperm can remain fertile in the cervix for up to several days after ejaculation [35]. Proteins in cervical secretions may protect sperm from premature capacitation. Once sperm capacitation occurs, the plasma membrane becomes unstable and easily undergoes acrosome reaction [13]. The sperm must reside at the right site, i.e., the fallopian tube ampulla, and undergo appropriate changes, i.e., successful capacitation, acrosome reaction, and fertilization, so that it can successfully fertilize [13, 45, 50]. Therefore, premature capacitation results in fertility loss. To preserve sperm fertility, secretory proteins in the lower reproductive tract may play an uncapacitation role and protect sperm from precocious capacitation.

Here, we demonstrated CST3 presence in cervical crypt and endometrium secretions during ovulation. Serum CST3 may enter the uterus by transudation because the endometrium permeability increases with elevated serum estrogen during the ovulation stage. CST3 with the ability to enhance motility may be helpful for sperm entering the upper female reproductive tract.

Ejaculated sperm cannot fertilize an egg after it has resided in the lower female reproductive tract for some duration; thus, the cervix and/or the uterus may secrete some proteins to stabilize the sperm plasma membrane and to thus prevent premature capacitation until sperm enters the fallopian tube. The cervical secretome, apart from CST3, may also contain other cervical secretory proteins with similar inhibitory effects on sperm capacitation. Cervical CST3 may be coupled with these secretory proteins to prevent sperm capacitation, and thus preserve sperm fertility until sperm reaches the fallopian tubes.

CST3 is extensively expressed in the endometrial epithelium, secretions of several animal species [36–38], and also in humans; progesterone was suggested to regulate CST3 expression levels [36–38]. Higher CST3 levels were detected in the endometrium during the mid- and late-secretory phase of the menstrual cycle, suggesting that CST3 expression in the human uterus may also be regulated by progesterone.

Because it inhibits protease’s activity, CST3 may be involved in endometrial remodeling and may also play a role in inhibiting sperm capacitation. Previous studies have demonstrated that several serine protease inhibitors, including HongrES1 [26], PEBP1 [27], and SERPINE2 [25], are involved in suppressing sperm capacitation.

This in vitro study makes it difficult to assess the effects of the female tract CST3 on sperm function in vivo; however, at least the potential effect of the female tract CST3 on human sperm function can be assessed. There may be one potential application for this study. In assisted reproductive technology, the fertilization rate because of intrauterine insemination (IUI) is < 20% [51]; however, the technique is less expensive and invasive than in vitro fertilization. If some protein factors with inhibitory effects on premature capacitation and acrosome reaction can be added, e.g., CST3, to protect sperm from precocious capacitation, IUI fertilization rates might improve.

## Conclusion

In conclusion, we have demonstrated that CST3 is expressed in epithelial cells lining the cervix, endometrium, fallopian tube, and in cervical mucus and uterine fluid, particularly during the late-proliferative phase around the time of ovulation. The cellular CST3 localization allows its interaction with sperm. It might bind to human sperm, enhance sperm motility, and potentially inhibit sperm capacitation.
